# The impact of reactive oxygen species in the development of cardiometabolic disorders: a review

**DOI:** 10.1186/s12944-021-01435-7

**Published:** 2021-02-27

**Authors:** Roland Akhigbe, Ayodeji Ajayi

**Affiliations:** 1 Department of Physiology, College of Medicine, Ladoke Akintola University of Technology, Ogbomoso, Oyo State Nigeria; 2 Reproductive Biology and Toxicology Research Laboratories, Oasis of Grace Hospital, Osogbo, Osun State Nigeria; 3 Department of Chemical Sciences, Kings University, Odeomu, Osun Nigeria

**Keywords:** Free radicals, OxLDL, PDGF, IGF, NOS uncoupling, ROS

## Abstract

Oxidative stress, an alteration in the balance between reactive oxygen species (ROS) generation and antioxidant buffering capacity, has been implicated in the pathogenesis of cardiometabolic disorders (CMD). At physiological levels, ROS functions as signalling mediators, regulates various physiological functions such as the growth, proliferation, and migration endothelial cells (EC) and smooth muscle cells (SMC); formation and development of new blood vessels; EC and SMC regulated death; vascular tone; host defence; and genomic stability. However, at excessive levels, it causes a deviation in the redox state, mediates the development of CMD. Multiple mechanisms account for the rise in the production of free radicals in the heart. These include mitochondrial dysfunction and uncoupling, increased fatty acid oxidation, exaggerated activity of nicotinamide adenine dinucleotide phosphate oxidase (NOX), reduced antioxidant capacity, and cardiac metabolic memory. The purpose of this study is to discuss the link between oxidative stress and the aetiopathogenesis of CMD and highlight associated mechanisms. Oxidative stress plays a vital role in the development of obesity and dyslipidaemia, insulin resistance and diabetes, hypertension via various mechanisms associated with ROS-led inflammatory response and endothelial dysfunction.

## Introduction

Cardiometabolic disorders (CMD) is a constellation of metabolic predisposing factors for atherosclerosis such as insulin resistance (IR) or diabetes mellitus (DM), systemic hypertension, central obesity, and dyslipidaemia [1]. They contribute to the global death rate and remain a public health challenge. There is a significant rise in the prevalence of CMD not only in high-resource countries but also in developing nations with emerging economies [2, 3]. Although, there are available data on the development of CMD and the mechanisms associated with its attendant complications, novel mechanisms are still revealed by recent studies in an attempt to open new therapeutic opportunities [4, 5]. Several studies have implicated oxidative stress in CMD development. It has been reported that cardio-tolerance to oxidative stress reduces with advancing age due to the antioxidant levels, particularly enzymatic antioxidants, contributing to the development of CMD [6]. Also, this has been linked with arterial thickening and atherosclerosis [7, 8]. This ensues in vascular endothelial damage and remodelling.

The high prevalence of CMD is a global phenomenon. The increase in the global prevalence is seemingly due to a parallel rise in the incidence of dietary and lifestyle changes, and cases of obesity [9]. There is an anticipated increase in these disorders due to projections of a greater incidence in future obesity cases [9]. The increased incidence of CMD among urban women when compared with their rural counterparts has been attributed to the global increase in urbanization and decline in physical exercise, especially in Africa [10]. A Nigerian study reported a prevalence of 18.0% and 10.0% in the semi-urban and rural community respectively and 34.7% and 24.7% respectively in a hypertensive population [11]. In Tunisia, Hosseinpanah *et al*. [12] documented a prevalence of 55.8% and 30.0% in women and men respectively. The higher prevalence in women was ascribed to the lower high-density lipoprotein (HDL), higher incidence of central obesity, and hypertension. According to the findings of Harzallah in Turkey [13], though female had a higher prevalence (39.6%) than the male counterpart (28%), the prevalence was similar in the urban (33.8%) and rural (33.9%) settings. In Qatar, prevalence rate of 26.5% was reported using Adult Treatment Panel III (ATP III) criteria and 33.7% using International Diabetes Foundation (IDF) criteria [14]. The observed incidence rose with advancing age and increasing body mass index, but reduced advancement in education and regular physical activity. In Lebanon, a prevalence of 31.2% was reported with men having a striking higher tendency [15]. Sibai [16] documented an age-adjusted prevalence of 37% in males in Saudi Arabia, with a higher prevalence in male (44%) than female (35.6%) [16]. In the USA in 2003/2004, using the National Cholesterol Education Program (NCEP)/ATP III criteria, about 34% people above 20 years old had CMD [17], with a marginal higher prevalence in male (35.1%) than female (32.6%). Although the prevalence of the disorder varies across geographic regions and age groups, about 25% of the adult European population was reported to have CMD [18].

This review highlights the role of ROS in the pathogenesis of CMD and discusses the associated mechanisms. This will shed more light to the pathogenesis of CMD and consequent open new therapeutic horizons.

## Methods

The present study reviewed all available data published in peer-reviewed journals up till date. Search was made using AJOL, DOAJ, Embase, Google Scholar, Pubmed/Pubmed Central, and Scopus databases using relevant key word searches like “Cardiometabolic disorders”, “metabolic disorders”, “Reactive oxygen species”, “ROS”, “oxidative stress”, “lipid peroxidation”, “Nitrosative stress”, and “Antioxidants and cardiometabolic disorders”. Papers published in peer-reviewed journals were included in this narrative review. Papers that did not adequately discuss details of the study were excluded from this review. Duplicated records were also excluded.

## Discussion

### Pathophysiology of CMD

CMD involve interplay of a cascade of pathophysiological events ensuing in a rise in IR, accumulation of free fatty acids (FFA) in the circulation, lipid and glucose dysmetabolism, and raised levels of adipokines and cytokines [19–21]. Since insulin controls adipose tissue lipid breakdown, the primary source of plasma (FFA), excess visceral fat causes IR with a resultant increase in lipids breakdown [22]. IR is further triggered by the increasing FFA concentrations via enhanced glucose dysregulation [23, 24]. These cumulate in ladening of fatty deposits in the blood vessels with resultant vasoconstriction, excessive fluid retention, and sustained rise in blood pressure [22].

A raised level of FFA does not just prevent the stimulation of glucose uptake in the muscle by insulin, [25] it also depresses the production of glucose in the liver [26], and enhances hepatic uptake of FFA. It causes increased production of VLDL and triglyceride (TG) in the liver [27–30], thus promoting TG transfer from VLDL to HDL and subsequent clearance of HDL [29, 30].

Inflammatory cytokines are generated by the adipose tissue and have the potential to trigger IR and adiponectin [31, 32]. Tumour necrosis factor-alpha (TNF-α) suppresses insulin signalling [33], interleukin-6 (IL-6) directly induces inflammation or enhances the release of hepatic C-reactive protein [34], while interleukin-8 (IL-8) activates neutrophil granulocytes. Adiponectin increases hepatic insulin sensitivity and oxidation of skeletal muscle glucose and fatty acid, and decreases glucose release [35–38]. This may infer that when there is abnormal plasma FFA concentration, the production of adipokines is raised while that of adiponectin is reduced.

Though obesity-associated excess visceral and/or intraperitoneal fat is strongly linked with IR [25, 26, 39, 40], whether or not intraperitoneal fat causes or is just associated with IR remains unclear. However, studies have suggested that fatty acids from lipolysis of intraperitoneal fat are a vital influential predisposing factor of IR since they are delivered to the liver directly through the portal vein [41]. Findings from the study of Havel *et al.* [42] among obese subjects revealed that lipolysis of the intraperitoneal fat accounts for 20% of FFA delivered to the liver and 15% delivered to skeletal muscle. Thus, intraperitoneal fat possibly contributes to hepatic IR, but unlikely to trigger skeletal muscle IR.

The pathogenesis of CMD involves a complex interplay between genetics and environmental factors. Also, epigenetic factors such as DNA methylation and histone modification are possibly key in the incidence of these disorders by mediating the effects of environmental exposures on the risk of development of CMD. The genetic factors are primarily hereditary and non-modifiable, while the environmental factors are modifiable. Race and advancing age are additional non-modifiable factors. Table 1 shows a list of some modifiable factors. Individuals at risk could be influenced by one or more hereditary and environmental factors which may worsen the pathogenic progress [22]. Thus, lifestyle modifications like diet and weight control, optimal exercise, cessation of cigarette smoking, and control of pollution and exposure to other mitochondrial toxins are beneficial factors that assuage CMD development.

**Table 1:**
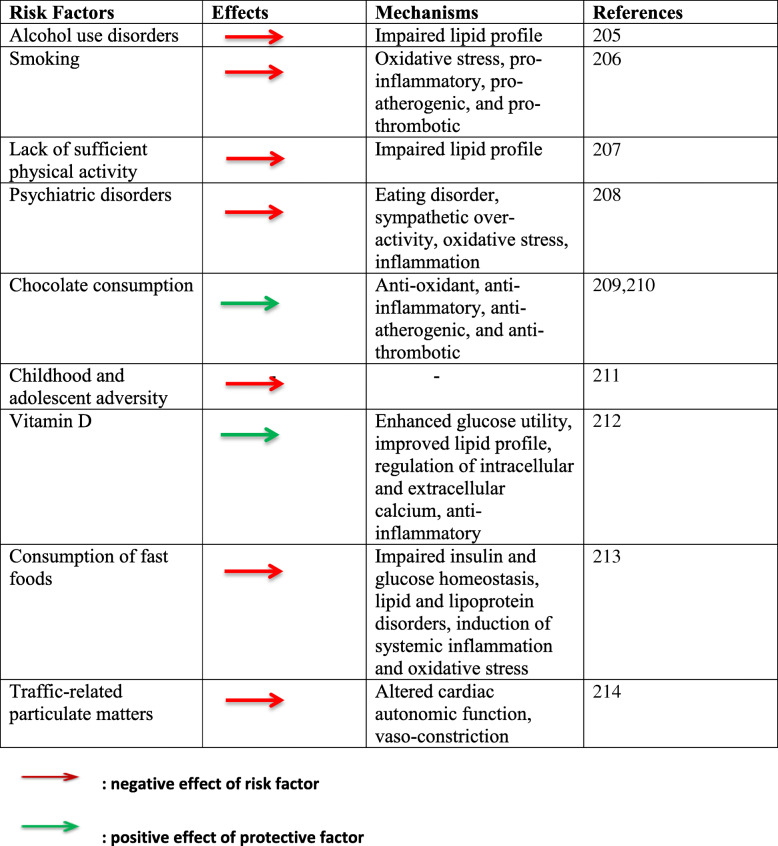
Modifiable protective and risk factors for cardiometabolic disorders

Epigenetics are alterations caused by developmental processes or environmental influences that do not modify the genetic code but influences the expression of the information encoded in the DNA [43]. Though there is limited evidence from genetic studies for a common genetic soil for CMD development, and contradicting relationships of genes and gene variants exists [44]. In order to promote early detection, prompt management and likely preventive strategies, a good an in-depth knowledge of the predisposing genetic factors influencing the development of CMD is important.

### Oxidative stress

**Fig. 1:**
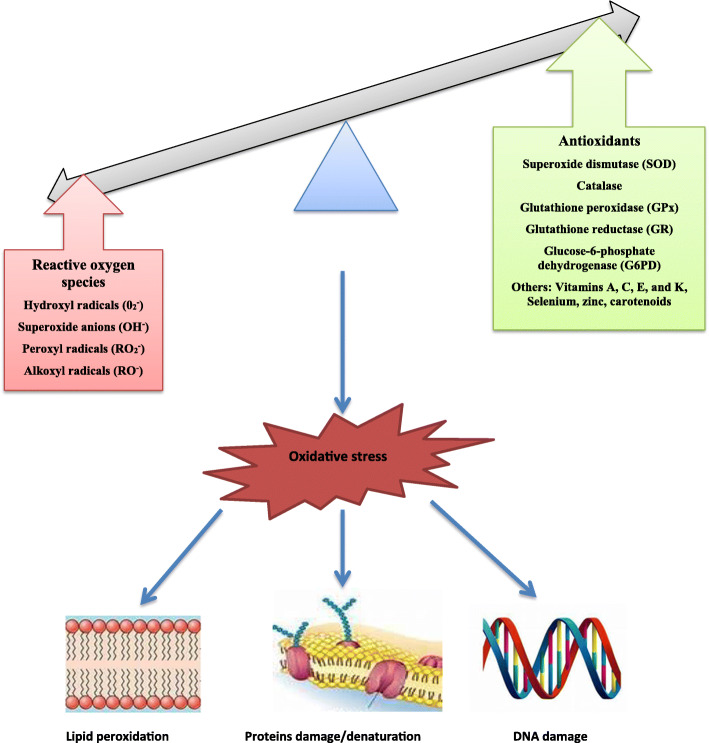
Oxidative stress resulting from an imbalance between ROS generation and antioxidant system and its consequences on cellular macromolecules

Oxidative stress (OS) is the presence of reactive oxygen species (ROS) in excess of the antioxidant buffering capacity. It can be described as an imbalance in ROS generation and antioxidant defence leading to accumulation of ROS. It is an alteration in the balance of prooxidant /antioxidant system in favour of prooxidant with attendant lethal effects with resultant damage to the cellular macromolecules (Fig. 1). In organisms, including humans, ROS and free radicals are produced during metabolic and immune system function. Molecular oxygen (O_2_) can unpair and leave free radicals which are highly unstable and reactive, leading to the formation of ROS [45, 46].

Free radicals are molecules with at least one unpaired electrons in their outer orbit. This makes them highly unstable and reactive with other molecules to produce more stable species [47]. Such radicals include Reactive Oxygen Species (ROS) and Reactive Nitrogen Species (RNS) [48]. Free radicals and oxidant species are involved in cellular and tissue dysfunction, and thus toxic [49]. Although low or moderate concentrations of these molecules play physiological roles such as signalling processes and defence mechanisms against infectious agents [49], when they are generated in excess, they lead to lipid, protein and DNA damage. This is a known fact, though stunning that ROS protects the cell against ROS damage by stimulating antioxidant responses and maintaining or re-establishing redox balance. It is noteworthy to state that in non-pathological states, ROS in low to moderate concentrations play a key homeostatic function in cellular and mitochondrial signaling and functionality; but, in excess concentrations when unchecked, it mediates oxidative cell and tissue damage. This can trigger positive feedback [50].

### Sources of cardiovascular ROS

#### Sources of ROS in cells

Vascular cells, including cardiac cells and neurons, generate ROS, thus triggering the incidence of CMD. Various enzyme systems such as cytochrome P450, the mitochondrial respiratory chain, xanthine oxidase (XO), uncoupled endothelial nitric oxide synthase (eNOS), heme oxygenase (HO), myeloperoxidase (MPO), lipoxygenase (LOX), cyclooxygenase (COX) and NADPH oxidases (NOX) generate ROS especially in a pathological state [51].

XO is a xanthine oxidoreductase that exists in two forms; xanthine dehydrogenase (XDH), which is the predominant form and can be irreversibly or reversibly converted into XO via proteolysis or the oxidation of cysteine residues respectively [52]. Its expression in the vascular endothelium is promoted by angiotensin II (Ang II) or oscillatory shear in a NOX-dependent manner [53].

The NOX family includes NOX1-5 and DUOX1-2. In humans, NOX2 NADPH oxidase seems to be the most important source of ROS generation [54–56]. NADPH oxidases are specific source of ROS because they produce ROS in a tightly-controlled manner unlike as in other sources where ROS are produced as a secondary metabolite [57]. Also, NADPH oxidases can also generate ROS from other enzyme systems [57].

Superoxide radical (O_2_^-^) is the first moiety that is produced by most of the enzyme systems, particularly NADPH oxidases. O_2_^-^ radical can undergo rapid dismutation to hydrogen peroxide (H_2_O_2)_, which is mediates most signaling effects of ROS [57].

#### ROS in the heart

**Fig. 2:**
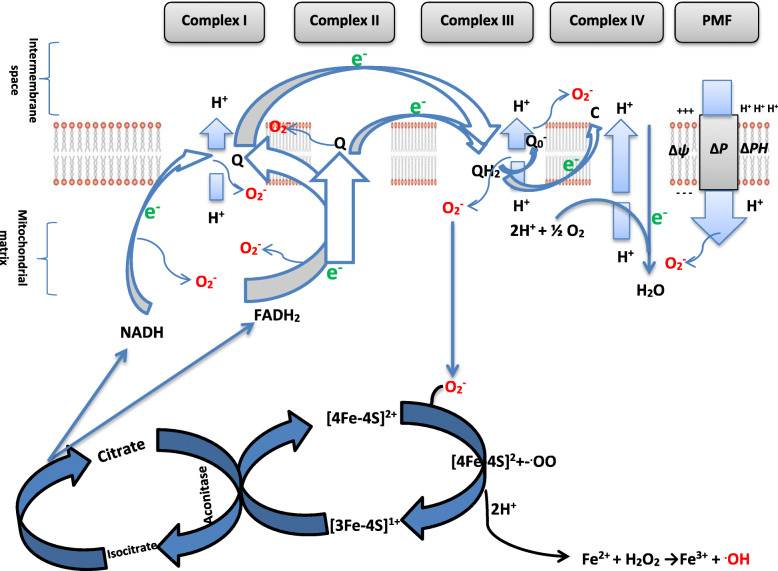
Generation of reactive oxygen species (ROS) by the mitochondria electron transport chain. Δ*p* : proton motive force, ΔΨ: membrane potential, Δ*pH* : proton gradient

Excessive ROS generation in the mitochondria has been shown in cardiomyocytes [58]. Mitochondria are the powerhouses of the living cells and produce energy primarily via oxidative phosphorylation. Also, mitochondria are the major source of ROS in the cardiovascular system [51, 58] (Fig. 2, Table 2). The aconitase of the Kreb’s cycle in the mitochondria matrix produces NADH and FADH2 which are oxidized for ATP production in the electron transport chain (ETC) located in the inner membrane of the mitochondria. The ETC mediates electron flow through series of electron carriers including complexes I, II, III and IV as well as ubiquinone and cytochrome c. As electron flows through the ETC, protons are translocated from the mitochondria matrix to the mitochondria inter-membrane space, thus creating an electrochemical potential gradient across the inner membrane. Generation of superoxide anion radicals in the mitochondria is mainly by electron leakage from the chain. Under physiological conditions, the oxygen tension in the mitochondria is low in state 4 respiration and oxygen consumption by the chain does not meet the demand of oxidative phosphorylation. Hence, a decrease in the rate of mitochondrial oxidative phosphorylation increases electron leakage from the ETC and conversely generation of superoxide anion radical.

**Table 2:** Enzymes and inhibitors of the electron transport chain (ETC)

Complex	Enzymes	Inhibitors
Complex I	NADH-ubiquinone oxidoreductase	Rotenone, Amobarbital (Amytal), Demerol, Piericidin,
Complex II	Succinate- ubiquinone oxidoreductase	Thenoyltrifluoroacetne (TTFA), Malonate oxaloacetate, Diazoxide
Complex III	Ubiquinone cytochrome-c oxidoreductase	Antimycin A, Myxothiazol, Stigmatellin
Complex IV	Cytochrome-c oxidase	Cyanide, Carbon monoxide, Azide, Hydrogen sulphide
Complex V	ATP synthase	Oligomycin A, Diyclo hexyl carbo dimide (DCCD)

XO generates O_2_^-^ as a secondary metabolite of purine catabolism. NOS uncoupling and subsequent O_2_^-^ generation have been linked with vascular endothelial dysfunction. Although, NOS primarily produces nitric oxide (NO), it may generate O_2_^-^ if it becomes uncoupled. Uncoupling of NOS is commonly seen when its co-factor, tetrahydrobiopterin (BH_4_), or its substrate, L-arginine, is deficient [59]. NADPH oxidases may also contribute to the generation of ROS by degrading BH_4_ via oxidation, thus causing NOS uncoupling [60], or activating xanthine oxidase [61].

Oxidative stress triggers apoptosis via several pathways activating enzymes involved in pro-apoptotic signalings, such as JNK, p38, ASK-1, and CaMKII [62] with resultant release of cytochrome-c. Though the excessive generation of ROS by NOX is deleterious, at minimal to moderate levels, H_2_O_2_ and O_2_^-^ produced by NOX, act as signalling molecules, thus mediating physiological responses [63].

#### ROS and the vasculature

ROS influence pathological and physiological processes in the vasculature. Predisposing factors to CMD like DM, obesity, hypertension, dyslipidaemia, and ageing result in vascular dysfunction partly through oxidative stress [64]. ROS is essential for the growth, proliferation, and migration of endothelial cells (ECs) and smooth muscle cells (SMCs), as well as angiogenesis, apoptosis of EC and SMC, vascular tone, host defence, and genomic stability [65]. OS does not only induce macromolecular damage, it also alters vascular redox-dependent signaling pathways [66]. ROS target signaling pathways such as mitogen activated protein kinases (MAPKs) which include extracellular signal-regulated kinases (ERK1/2), p38 and c-Jun N-terminal kinases [67]. It also targets serine/threonine kinase Akt/protein kinase B, epidermal growth factors (EGF), and platelet-derived growth factors (PDGF). In oxidative stress, ROS triggers endothelial dysfunction via disruption of vasoprotective NO signaling [68].

NO is a vasodilator that acts via cGMP which is produced by SMCs. NO inhibits platelet adhesion and aggregation, thus acting as an anti-atherogenic factor. It also prevents leukocyte-endothelial interactions and MC proliferation [69]. O_2_^-^ rapidly reacts with NO to produce peroxynitrite (ONOO^-^), which is also a potent oxidant [70]. ONOO^-^ formation occurs more rapidly than superoxide dismutase (SOD)-dependent dismutation of O_2_^-^ [71]. The formed ONOO^-^ induces BH_4_ oxidation with resultant eNOS uncoupling [72] thus converting eNOS into a pro-oxidant [73]. Hence, ONOO^-^ formation depletes NO concentration and also induces eNOS uncoupling.

ROS also triggers the structure of the inflammasome, IL-1β, and IL-8 via the activation of caspase-1 [74]. This pathway is vital in atherosclerosis.

As highlighted previously, NADPH oxidase, XO, and uncoupled eNOS, including lipoxygenase, cyclooxygenase, and cytochrome P450 monooxygenase are key enzymes that generate ROS in the cells as well as in the vascular wall. ROS are involved in the structural modification of the vascular wall thickness and lumen diameter [75]. This is consequent to the passive adaptation to chronic changes in hemodynamics and neuro-humoral factors such as angiotensin II and ROS [75]. This vascular remodelling could be inward eutrophic or hypertrophic, and play a role in the development of hypertension. The inward eutrophic remodelling involves reduction in the size of the lumen, media thickening, improved media to lumen ratio, and little alteration in the cross-sectional area of the media [76], while the hypertrophic remodelling involves an enhancement in the cross-sectional area of the vascular wall, size of the cell and accumulation of ECM proteins like collagen and fibronectin [77].

#### Vascular ROS scavenging

##### Mitochondria as the primary source of ROS

CMD is accompanied by an imbalance in vascular ROS generation and scavenging. The primary defense against vascular ROS is discussed below.

##### Superoxide dismutase

In humans, SOD1, SOD2, and SOD3 are the three known SOD isoforms. SOD1 (copper, zinc [Cu-Zn]-SOD) is located in the cytoplasm and mitochondrial intermembrane space, SOD2 (Mn-SOD) is located in the mitochondrial matrix, while SOD3 (extracellular [EC]-SOD) is located in the extracellular space [78]. SOD dismutates O_2_^-^ to H_2_O_2_ and oxygen, hence prevents the inactivation of NO. However, high concentrations of the secondary metabolites of the dismutation may cross cellular membranes to generate pro-atherogenic molecules [78–81]. The effect of SOD is dose-dependent.

##### Catalase

Catalase decomposes H_2_O_2_ to oxygen and water. Up-regulation of this enzymatic antioxidant inhibits atherosclerosis [80] and impairs angiotensin II-mediated aortic wall hypertrophy [82].

##### Glutathione peroxidase

Glutathione peroxidase (GPx) catalyzes the reduction of H_2_O_2_ to water, and lipid peroxides to their alcohols [83, 84]. Although there are 8 isoforms of GPx, GPx1 and GPx4 seem to be the most studied. GPx1 is found in many cell types, and its deficiency has been linked with atherosclerosis [85, 86]. On the other hand, GPx4 is expressed in the endoplasmic reticulum (ER), cytoplasm, mitochondria, and plasma membrane. GPx4 prevents atherogenesis by impairing lipid peroxidation and the sensitivity of vascular cell to oxidized lipids [87].

##### Paraoxonase

Paraoxonases (PON) include PON-1, PON-2, and PON-3. PON exhibit anti-atherogenic properties, possibly via inhibition of oxidative stress [88]. PON-1 is primarily secreted by the liver [89]. PON-1 prevents the peroxidation of HDL and LDL, breaks down cholesteryl esters and lipoproteins seen in oxidized lipoproteins, and also prevents OS, inflammation, and monocyte attraction via blunting myeloperoxidase-induced ROS generation. PON-2, which is expressed in the ER and mitochondrial membranes, exerts its effects on the vascular cells [90, 91]. PON-3, which is located in the serum and cells [92], prevents atherogenesis [93, 94].

##### Heme oxygenase

HO catalyzes degradation of heme to carbon monoxide (CO), biliverdin, and free ferrous iron [95]. HO exists in three isoforms; the inducible (HO-1), constitutive (HO-2), an enzymatically inactive (HO-3) forms [95]. OS, hypoxia, and some cytokines stimulate the upregulation of the inducible isoform, which are key in impairing vascular remodelling and atherosclerosis [95]. Although at moderate concentrations, the CO produced by HO has anti-inflammatory, antiproliferative, and vasodilatory activities, it is toxic at a very high concentration [96]. Biliverdin is a pigment that scavenges radicals and also blunts the effect of NOX [97].

##### Thioredoxin

Thioredoxin (Trx) is an enzymatic antioxidant that is located in the ECs, SMCs, and fibroblasts [98]. It is vasoprotective and reverses age-related arterial stiffness and raised blood pressure via enhancement of vascular redox and restoration of the function of eNOS [98].

##### Non-enzymatic antioxidants

Bilirubin, uric acid, glutathione, exogenous substances like vitamins (mainly vitamins C and E) and polyphenols, contribute to the antioxidant defence system [99]. Bilirubin and uric acid scavenge extracellular radicals, while glutathione modifies the intracellular redox state [99]. Vitamin C (ascorbic acid) scavenges several oxidative/nitrogen species, stabilizes BH4 and eNOs, and restores vitamin E from its radical state (tocopheroxyl radical) [100]. α-tocopherol is the principal member of vitamin E with antioxidant property. Sources of polyphenolic antioxidants include food such as vegetables and cocoa and beverages. Polyphenolics impair NADPH oxidases activities [101, 102].

Micronutrients like selenium, copper, zinc, iron, and calcium also contribute to the antioxidant buffering capacity. Selenium protects against oxidative DNA damage. It acts via selenoenzymes-mediated mechanism such as GPx [103]. As selenium serves as a co-factor of GPx, copper and zinc are co-factors of SOD, while the iron is a co-factor of catalase. Thus, these elements influence the activities of the respective enzymatic antioxidants. Note worthily, iron and copper may act as pro-oxidants by catalyzing the production of hydroxyl (OH) radicals from O_2_^-^ and H_2_O_2_ [104, 105]. Although calcium is critical in excitation-contraction coupling, it also plays vital physiological roles like the regulation of gene expression and cellular energetics [106–110].

### Biomarkers of oxidative stress

Oxidative stress has been shown to be a key player in various diseases including cardiometabolic disorders. A wide range of methods have been developed and employed to measure the nature and extent of oxidative stress ranging from oxidation of lipids to free amino acids and proteins, and DNA. Although diverse oxidative stress biomarkers are available as predictors of various diseases, the specificity of each seems to be yet established. Available biomarkers of oxidative stress have been summarized in Table 3.

**Table 3:** Biomarkers of oxidative stress

Markers of oxidative stress	
Lipid oxidation	Malondialdehyde (MDA), 4-hydroxy-2-nonenal (4-HNE), F2-isoprostanes, isolevuglandins, acrolein, crotonaldehyde, and methylglyoxal.
Protein/amino acid oxidation	Protein carbonyls, advanced glycation endproducts (AGEs), advanced lipoxigenation end products (ALEs), advanced oxidation protein products (AOPP), 3-nitrotyrosine, ischemia-modified albumin (IMA), oxidized low density lipoprotein (oxLDL)
DNA oxidation	8-oxo-2’ - deoxyguanosine (8-oxo-dG; 8OHdG), 5-chlorocytosine, 5-chlorouracil,
Markers of ROS generation	
Enzymatic	Xanthine oxidase (XO), myeloperoxidase (MPO), nicotinamide adenine dinucleotide phosphate oxidase (NOX), nitric oxide synthase (NOS)
Non-enzymatic	Uric acid
ROS-regulated transcription factors	Nuclear factor kappa-light-chain-enhancer of activated B cells (NF-kB)
Markers of antioxidant defense	
Enzymatic	Superoxide dismutase (SOD), catalase (CAT), glutathione peroxidase (GPx), glutathione reductase (GR), glutathione S-transferase (GST), glucose -6- phosphate dehydrogenase (G6PD), Protein thiol-disulfide oxidoreductases [thioredoxin (Trx) and peroxiredoxins (Prxs)].
Non-enzymatic	Ascorbic acid, α-tocopherol, β-carotene, polyphenols, bilirubin, albumin, ceruloplasmin, ferritin, glutathione (GSH).
ROS-regulated transcription factors	Nuclear factor (erythroid-derived 2)-like 2 (Nrf-2)
Others	Asymmetric dimethyl L-arginine (ADMA)

### Oxidative stress and CMD

#### Oxidative stress and obesity

**Fig. 3:**
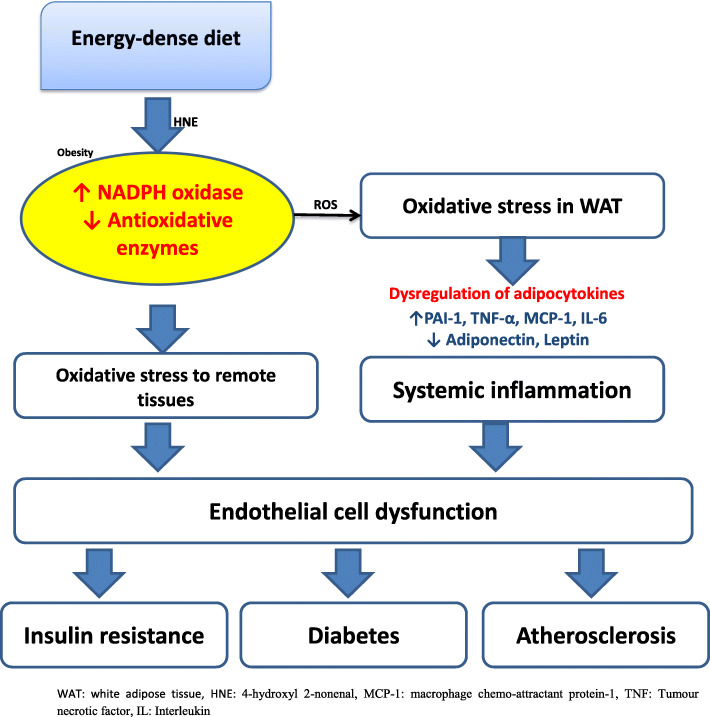
The role of oxidative stress in the development of obesity and cardiometabolic disorders. This illustration is a modification of the working model illustrating how increased ROS production in accumulated fat contributes to metabolic syndrome by Furukawa et al. [115]

Emerging evidences implicating OS as the soil for the initiation and progression of dyslipidaemia, obesity, IR, DM, hypertension and atherosclerosis exit. Obesity is the primary causal component of CMD [111–114] (Fig. 3). Consumption of an energy-dense meal is linked to a significant increase in the concentrations of 4-hydroxyl 2-nonenal (HNE) [116], thus is essential in the incidence of obesity. Notably, Johnson et al. [117] have reported a reduced HNE level of HNE in obese individuals on calorie restriction. This underscores the essence of OS via HNE in incident obesity. ROS generation rises in parallel with fat adipocyte fat accumulation, and the increase in the level of FFAs also stimulates adipocyte ROS generation via NADPH oxidase activation and decline in enzymatic antioxidant expression [115]. In the presence of OS in adipocytes, anti-inflammatory adiponectin level falls [118, 119], while pro-inflammatory adipocytokines concentration rises [115, 120, 121]. Dysregulation of adipocytokines is vital in the development of obesity-associated metabolic disorder. Raised adipocyte generation of PAI-1, MCP-1, and TNF-α is important in the pathogenesis of thrombosis [122], and IR [123, 124]. Since adiponectin increases cellular sensitivity to insulin [37, 125–127] and also possesses anti-atherogenic effects [128–130], a marked reduction in the circulatory concentrations of adiponectin results in IR and atherosclerosis via systemic inflammation [115]. HNE up-regulates the expression of inducible cyclooxygenase (COX-2) and PAI-1 [131] and down-regulates the expression of adiponectin [119, 132]. Dysregulation of adipocytes results in systemic inflammation; this as well as increased adipocyte ROS generation promotes endothelial dysfunction. This is key in the development of IR, DM and atherosclerosis. Interestingly, renin-angiotensin-aldosterone system (RAAS) which plays an important role in blood pressure and volume regulation, has also been demonstrated to trigger adipocyte ROS generation [133].

#### Oxidative stress and IR/diabetes

Following systemic inflammation, oxidative damage to the endothelial cells causes impaired glucose uptake and utilization by hepatocytes and skeletal myocytes. Activation of NOX via RAAS increases endothelial ROS generation [134, 135]. This is dependent on angiotensin II type-1 and mineralocorticoid receptors [136]. This cascade of events continues and causes a transition from IR to DM type II [137]. Oxidative injury to the endothelium reduces the circulatory level of NO due to the decline in its synthesis by uncoupling eNOS via ROS-induced oxidation and depletion of BH4 [138, 139]. Increased generation of ONOO^-^ via coupling of NO to superoxide also contributes to NO depletion [140]. The generated ONOO^-^ is very reactive and leads to endothelial cell death [140, 141] which also impairs endothelial NO generation. It is a known fact that eNOS-derived NO is essential in angiogenesis by enhancing vascular endothelial growth factors and up-regulating the recruitment of endothelial progenitor cells from the bone marrow [142, 143]. Hence ROS-induced decline in circulatory NO secondary to endothelial dysfunction impairs the growth of the capillary network and blood flow regulation and subsequent diminution of microcirculation in metabolically active tissues and dysregulations of glucose and dyslipidaemia.

**Fig. 4:**
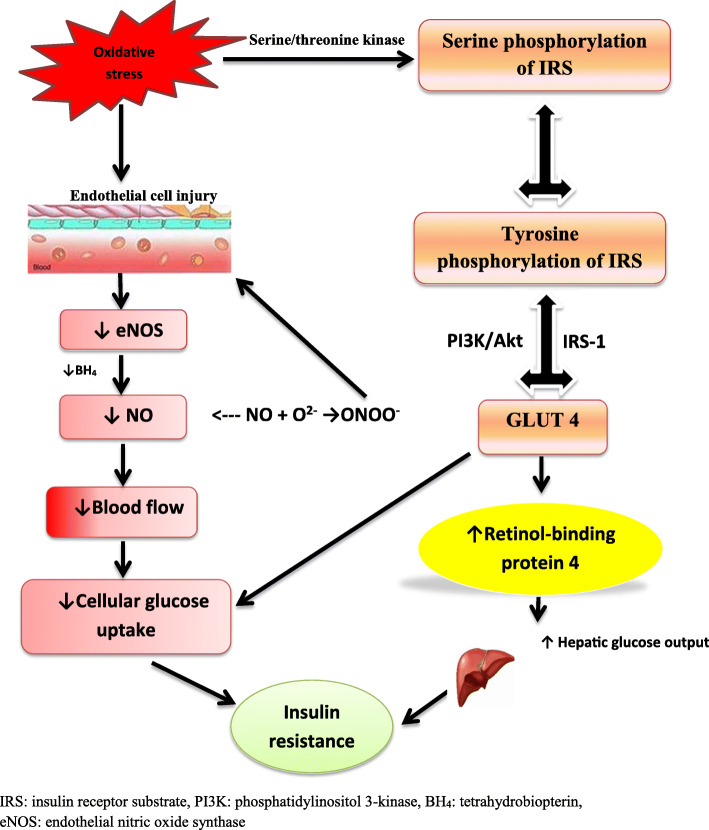
The role of oxidative stress in the pathogenesis of insulin resistance/type II diabetes. This illustration is a modification of the mechanism illustrating the role of oxidative stress to adipocytes in insulin resistance by Otani H [137].

Studies have implicated OS in IR and DM through insulin signaling, insulin-induced GLUT 4 translocation and glucose uptake via insulin receptor substrate (IRS) phosphorylation, MARK activation and ER stress. Activation of serine/threonine kinase cascade stimulates serine phosphorylation of IRS that in turn impairs tyrosine phosphorylation thus possibly enhancing IRS degradation [144], resulting to alteration of glucose uptake signalling pathways by GLUT4 via IRS-1 and phosphatidylinositol 3-kinase (PI3K)/Akt [137]. Impairment of glucose uptake through this pathway exerts a further negative effect on IR. Adipocytes, which act as glucose sensors [145], senses impaired GLUT4-mediated glucose uptake and release adipocytokines (such as retinol-binding protein 4, RBP 4) to prevent glucose uptake by skeletal muscle and improve hepatic glucose output through insulin signalling blockade [146]. This sums up to raised plasma level of glucose. Hence OS mediates the development of IR and DM type II via downregulation of circulatory NO bioavailability and GLUT 4 expression in adipocytes (Fig. 4).

#### Oxidative stress and hypertension

**Fig. 5:**
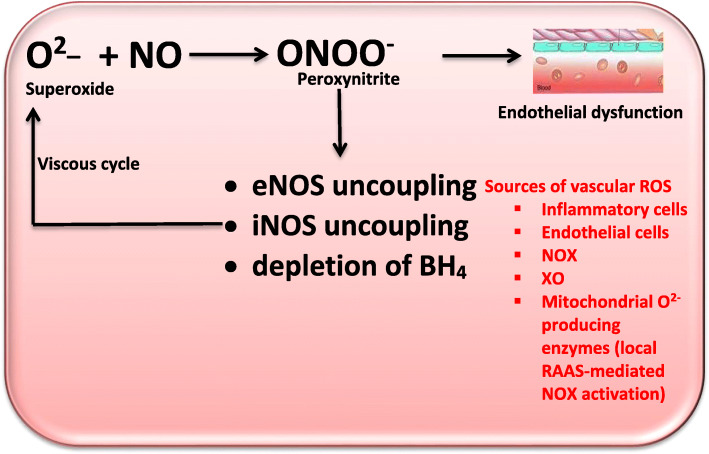
The role of oxidative stress in the pathogenesis of hypertension. XO: xanthine oxidase, NOX: nicotinamide adenine dinucleotide phosphate oxidase, eNOS: endothelial nitric oxide synthase, OxLDL: Oxidized LDL, ROS: reactive oxygen species, PDGF: Platelet-derived growth factor, SMC: smooth muscle cells, MF: Myofibrils

The role of NO in the pathophysiology of hypertension has well clearly described. NO enhances angiogenesis and blood supply, thus regulates blood pressure. NO may react with O^-^ to increase the generation of ONOO^-^, which break down to form hydroxyl radical [147, 148]. The generated ONOO^-^ causes eNOS uncoupling, iNOS uncoupling, and BH_4_ depletion [137]. Under physiological conditions, electrons are transferred from a heme group in the oxygenase domain to L-arginine by eNOS. This leads to the generation of L-citrulline and NO [149]. However, when there is a depletion of NO, may be due to depletion of L-arginine, a substrate of NO, or BH_4_, a co-factor in NO production, eNOS switches to an uncoupled state from a coupled state. This leads to the reduction of oxygen by electrons from the heme group with subsequent production of superoxide radical (O_2_^-)^ [150]. This radical reacts with NO to further deplete NO, leading to endothelial dysfunction and rise peripheral resistance, thus causing a sustained rise in blood pressure. Depletion of L-arginine is associated with exaggerated arginase II activity. iNOS expression is also up-regulated in endothelial dysfunction [137]. Although iNOS generates NO, due to oxidative stress-induced BH_4_ depletion and iNOS uncoupling, iNOS uncoupling enhances oxidative stress. Also, it leads to a vicious cycle of endothelial dysfunction and persistent rise in blood pressure (Fig. 5). In addition, endogenous eNOS inhibitor, asymmetric dimethyl-L-arginine (ADMA) may contribute to eNOS uncoupling. Oxidative stress has been demonstrated to enhance the activity of protein arginine N-methyltransferase (PRMT) and reduce the activity of dimethylarginine dimethylaminohydrolase (DDAH), an ADMA-degrading enzyme, thus resulting in a rise in ADMA concentrations [69, 151, 152]. The rise in ADMA levels may inhibit NO synthesis by eNOS or even lead to eNOS uncoupling [69, 151]. Note worthily, BH_4_ depletion-driven eNOS uncoupling may rather be secondary to the oxidation of zinc-thiolate cluster of eNOS. Exposure of isolated eNOS to ONOO^-^ causes disruption of the zinc-thiolate cluster of the enzyme [153]. Since the Cys99 in this cluster is essential for BH_4_ binding, oxidation of this cluster will disrupt the BH_4_ binding site of the enzyme; an state similar to depletion of BH_4._

Local RAAS activation plays an integral role in the complex cascades that contribute to endothelial dysfunction. Studies have shown that the accumulation of visceral fat and raised OS and inflammatory response in adipose tissue enhance the release of components of adipose RAAS. Animal studies have demonstrated up-regulation of angiotensinogen in fatty tissue in obesity. This is strongly linked with hypertension [154]. Angiotensinogen converts angiotensin I to angiotensin II, which exerts its effects via angiotensin II type 1 receptor (AT_1_R) [149]. In the zona glomerulosa of the adrenal cortex, activation of AT_1_R triggers the release of mineralocorticoids [149], which elevate blood pressure primarily via stimulation of sodium reabsorption and expansion of plasma volume, and secondarily via non-genomic mineralocorticoid receptor (MR)-mediated actions [155]. In non-adrenal tissues, activation of AT_1_R triggers ROS generation with resultant impairment of insulin signaling, as well as proliferative and inflammatory responses [156]. This penultimately leads to endothelial dysfunction and hypertension. It is worthy to note that mineralocorticoids such as aldosterone and deoxycorticosterone acetate have been reported to activate NADPH, trigger oxidative stress and release superoxides [157, 158].

XO is a hypoxia-inducible enzyme, which is found in vascular smooth muscle cells (VSMCs) and vascular endothelial cells. XO catalyzes superoxide production [149]. Mervaala and colleagues [159] documented that rodents with over-expressed human renin and angiotensinogen genes have raised XO activity with endothelial dysfunction and hypertension. Experimental studies have also demonstrated increased renal XO activity in salt-fed spontaneously hypertensive rats [160].

Membrane-bound vascular-derived NOX enzymatic complex can be activated by angiotensin II and aldosterone even at low concentrations [161]. Activation of this system is a major source of ROS, which leads to NO depletion and endothelial dysfunction. Mounting number of studies has implicated the crosstalk between NOX and mitochondria with incident eNOS dysregulation/uncoupling and endothelial dysfunction. It has been established that Ang II stimulates mitochondrial ROS (mtROS) formation and opening of the mitochondrial permeability transition pore (mPTP) with resultant leakage of the generated mtROS to the cytosol [162, 163]. This activates the p38 MAPK and JNK signaling with subsequent activation of NOX [162, 163]. Also, NOX could be activated through cSrc-dependent phosphorylation of p47phox, which is triggered by Ang II. Ang II-dependent NOX causes mitochondrial dysfunction with consequent mtROS formation [164, 165]. This cascade of events leads to a robust accumulation of mtROS formation with cardiovascular implications. mtROS-driven phagocytic NOX activation triggers immune cell infiltration and aggravates Ang II-mediated eNOS uncoupling [166], reduced circulatory NO and endothelial dysfunction.

Although enzymatic superoxide dismutase scavenges the superoxides that are produced in the mitochondria during oxidative phosphorylation, this mechanism may be overwhelmed when ROS generation is exaggerated. This leads to mitochondrial DNA damage and endothelial injury [167].

#### Oxidative stress and atherosclerosis

**Fig. 6:**
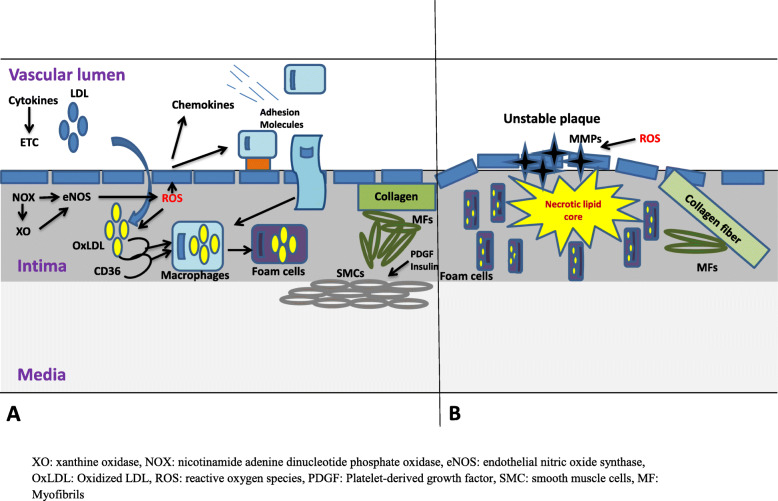
The role of oxidative stress in the pathogenesis of atherosclerosis – early phase (**a**) and late phase (**b**). This illustration is culled from the illustration of the role of oxidative stress to adipocytes in atherosclerosis by Otani H [137].

Atherosclerosis is the common clinical disorder that results from obesity, IR and DM, and hypertension (Fig. 6). It begins with the formation of atheromatous plaque, which is triggered by endothelial ROS generation and accumulation of LDL in the intima. LDL consists of an ApoB protein molecule, triglycerol, cholesterol and its esters, phospholipids, and vitamin E [168]. The presence of TG enhances the influx and accumulation of LDL in the tunica intima, where it is oxidized by ROS, and picked up by macrophages via scavenger receptor (SR) CD36 to produce foam cells [169–171]. Other SRs such as SR-AI, SR-AII, MARCO, and SRCL (class A), SR-BI (class B), CD68 (Class D), LOX-1 (class E), SREC-1 (Class F), and SR-PSOX/CXCL16 (Class G) have been reported [172].

Oxidized LDL (OxLDL) is cytotoxic to atherosclerosis-related cells such as T-cells, macrophages, ECs, and SMCs [173] via OxLDL-derived lipid peroxides and hydroperoxides [174]. High concentrations of OxLDL activates caspase 3 in a Fas-independent manner, thus causing apoptosis with characteristic DNA fragmentation [168]; although, caspases 6, 8 and 9 may also be involved. Besides, OxLDL simultaneously triggers necrosis via ROS [168]. Although OxLDL suppresses nuclear factor-kappa B (NF-kB) in long-term, it activates it in short-term in ECs, SMCs, and macrophages [175–177]. OxLDL-induced NF-kB activation is via LOX-1. Binding of OxLDL to LOX-1 promotes the production of O^-^ and H_2_O_2_, as well as activation of NF-kB via p38 MAP kinase//P13K/ERK1/2 signaling pathway [178, 179], thus eliciting an inflammation in the endothelial cells. NF-kB regulates the expression of MCP-1, P-selectin, E-selectin, ICAM-1, and VCAM-1 [180].

Recruitment of monocyte-macrophage into the intima is regulated by adhesion molecules, integrins, selectins, and chemokines such as monocyte chemo-attractant protein-1 (MCP-1) [181, 182]. ROS does not just oxidize OxLDL; they also up-regulate MCP-1 and other molecules that are responsible for monocyte-macrophage recruitment. Although, SMCs and ECs synthesize MCP-1, SMCs also moves to the tunica intima from the tunica media, where they differentiate into myofibroblasts and increase in the presence of PDGF [183, 184] and insulin via ROS-dependent phosphorylation of serine residues of IRS-1 [185, 186]. These myofibroblasts are responsible for collagen synthesis, which causes intima thickening.

Enhanced inflammatory response and OS promote apoptosis of the foam cells and necrotic lipid core formation [187, 188]. The necrotic lipid core is covered with a collagen fibre-enriched fibrous cup which is lyzed by activation of matrix metalloproteinases (MMP) in the presence of ROS [189, 190] generating an advanced atheromatous plaque called unstable plaque. This narrows the lumen of the artery and raises the intraluminal pressure.

It has been established that coronary artery disease progresses faster in diabetic patients. There are mounting evidence that suggestive that adiposity and impaired glycaemia promote the development of ischaemic heart disease (IHD) even in non-diabetic individual [191, 192]. Although insulin-treated diabetes has been shown to be an independent predictor of late and repeat coronary revascularization [193], study of Komatsu et al. [194] revealed that IR in the general population treated with percutaneous coronary intervention (PCI) enhanced restenosis due to continuous neointimal growth after the first generation drug eluting stent (DES) implantation. Sasso and his colleagues [192] in a prospective longitudinal observational study demonstrated the role of IR and cytokines in the incident of IHD in normoglycaemic subjects. Findings of their study showed that adiponectin levels were independently associated with restenosis; and HOMA-IR and adiponectin were independently associated with de novo IHD and overall new PCI.

### Cardiac metabolic memory

Even when glucose level has been restored, chronic rise in glucose concentration as seen in DM stimulates metabolic alterations that modify tissue homeostasis. This is called metabolic memory [195]. Epigenetics is essential in establishing cardiac metabolic memory. Chronic epigenetic effects like histone and DNA methylations are quite stable and may be inherited as a memory by offspring cells [196]. In addition, maternal nutrition as well as in utero exposure may trigger developmental programming which may also be transferred to progenies, thus triggering disorders like CMD [197]. Hyperglycemia may trigger long-term inflammatory and oxidative stress pathways. This is accompanied by resultant persistent or possibly permanent modifications [198]. Experimental study demonstrates hyperglycemia-dependent ROS as a primary trigger of endothelial glycemic memory [199]. Studies have revealed that hyperglycemia stimulates a reversible increase in the concentrations of IL-6 and decline in histone-3 methylation at the IL-6 promoter in cardiomyocytes [200]. This infers that the raised inflammatory gene expression in cardiomyocytes observed in hyperglycemia is secondary to impaired repressive epigenetic histone modifications [200]. It is not unlikely that hyperglycemia-stimulated mitochondrial dysfunction and apoptosis account for cardiac metabolic memory [200]. Also, hyperglycemia triggers developmental control of insulin-like growth factor-1 (IGF-1) receptor in cardiac muscle cells [201]. Human studies have revealed that a rise in adipose tissue accumulation in obesity is linked to enhanced methylation at the hypoxia-inducible factor 3A (HIF3A) locus in blood cells and adipose tissue, but not in the skin [202]. Metabolic intermediates from catabolism of macromolecules serve as co-factors for chromatin-modifying enzymes [203–214].

## Conclusion and future perspectives

Oxidative stress, through a complex cascade, is essential in the development of CMD. Increasing evidence has demonstrated that ROS via various pathways triggers systemic inflammation and endothelial cell dysfunction through several mechanisms, such as mitochondrial dysfunction and uncoupling, raised FAO, up-regulation of NOX activity, impaired antioxidant capacity, and cardiac metabolic memory. Although more studies aimed at demonstrating other associated pathogenesis of CMD is important, a good understanding of the link between oxidative stress and CMD opens new therapeutic horizons in the management of CMD by targeting one or more specific pathways in the pathophysiology.

## Data Availability

Not applicable

## Abbreviations

CMD: CMD
HDL: high-density lipoprotein
LDL: low-density lipoprotein
OxLDL: Oxidized LDL
TG: triglycerides
VLDL: very-low-density lipoprotein
ATP III: Adult Treatment Panel III
IDF: International Diabetes Foundation
(NCEP): National Cholesterol Education Program
TNF-α: tumour necrosis factor-alpha
IL: interleukin
MCP-1: macrophage chemo-attractant protein 1
ROS: reactive oxygen species
RNS: reactive nitrogen species
XO: xanthine oxidase
eNOS: uncoupled endothelial nitric oxide synthase
HO: heme oxygenase
XDH: xanthine dehydrogenase
Ang II: angiotensin II
GDP: guanosine diphosphate
PI (3) P: phosphatidylinositol 3-phosphate
BH_4_: tetrahydrobiopterin
ECs: endothelial cells
SMCs: smooth muscle cells
GTP: guanosine-5’-triphosphate
MAPKs: mitogen-activated protein kinases
ERK: extracellular signal-regulated kinases
NO: nitric oxide
NOX: nicotinamide adenine dinucleotide phosphate oxidase
O_2_^-^: superoxide anion radicals
ONOO^-^: peroxynitrite
ECM: extracellular matrix
HIF: hypoxia-inducible factor
SOD: superoxide dismutase
H_2_O_2_: hydrogen peroxide
GPx: glutathione peroxidase
PON: paraoxonases
CO: carbon monoxide
Trx: thioredoxin
SR: sarcoplasmic reticulum
SERCA: sarcoplasmic reticulum ATPase
HNE: 4-hydroxyl 2-nonenal
COX: cyclooxygenase
RAAS: renin-angiotensin-aldosterone system
IRS: insulin receptor substrate
PI3K: phosphatidylinositol 3-kinase
RBP: retinol-binding protein
GLUT: glucose uptake transporter
AT_1_R: angiotensin II type 1 receptor
VSMCs: vascular smooth muscle cells
NF-kB: nuclear factor-kappa B
LOX: lipooxygenase
VCAM: ICAM
PDGF: platelet-derived growth factor
SMC: smooth muscle cells
MMP: matrix metalloproteinases
IGF-1: insulin-like growth factor-1
FAO: Fatty acid oxidation
PCI: percutaneous coronary intervention
DES: drug eluting stent
